# Anticonvulsant and Neuroprotective Activities of* Phragmanthera austroarabica* Extract in Pentylenetetrazole-Kindled Mice

**DOI:** 10.1155/2017/5148219

**Published:** 2017-03-30

**Authors:** Hibah M. Aldawsari, Basma G. Eid, Thikrayat Neamatallah, Sawsan A. Zaitone, Jihan M. Badr

**Affiliations:** ^1^Department of Pharmaceutics, Faculty of Pharmacy, King Abdulaziz University, Jeddah 21589, Saudi Arabia; ^2^Department of Pharmacology and Toxicology, Faculty of Pharmacy, King Abdulaziz University, Jeddah 21589, Saudi Arabia; ^3^Department of Pharmacology and Toxicology, Faculty of Pharmacy, Suez Canal University, Ismailia 41522, Egypt; ^4^Department of Natural Products and Alternative Medicine, Faculty of Pharmacy, King Abdulaziz University, Jeddah 21589, Saudi Arabia; ^5^Department of Pharmacognosy, Faculty of Pharmacy, Suez Canal University, Ismailia 41522, Egypt

## Abstract

Anticonvulsant and neuroprotective activity of* Phragmanthera austroarabica* extract were tested in pentylenetetrazole-kindled mice. All the chemical constituents of the plant extract were identified. Additionally, the extract was standardized and proved to contain total phenolic contents equal to 379.92 ± 1.32 mg gallic acid equivalents/g dry plant extract. Induction of kindling was achieved by repeated intraperitoneal administration of pentylenetetrazole (35 mg/kg) twice weekly. Male albino mice were given* P*.* austroarabica* extract (200, 400, or 800 mg/kg). The two higher doses (400 or 800 mg/kg) of the extract significantly caused notable reduction in seizure activity and hippocampal malondialdehyde level compared to pentylenetetrazole control group. The highest dose enhanced cortical GSH level and showed intact DNA in the laddering assay. Upon studying the neuroprotective effect, mice treated with the higher dose of the extract demonstrated an improvement in the percent of surviving neurons in the cortex and hippocampus. We concluded that* P. austroarabica* extract ameliorated seizure activity and protected cortical and hippocampal neurons against pentylenetetrazole-induced kindling in mice.

## 1. Introduction

A crucial challenge is to discover the causes of the variety of neurodegenerative diseases. This will allow the prevention or slowing of the resultant disorders. In many neurodegenerative diseases, oxidative stress is considered as a common factor. Additionally, it is recognized as the most probably proposed mechanism for degenerative processes related to age [1, 2]. A considerable number of previous studies illustrated a strong evidence linking oxidative stress to a number of neuronal diseases as Alzheimer's disease [3, 4], Parkinson's disease [5, 6], and multiple sclerosis [7, 8]. Damage of the neuronal cells is mediated by reactive oxygen species. Accumulation reactive oxygen species causes peroxidation of lipid and damage of DNA and protein and finally may lead to cell death [9]. Antioxidants have the ability of inhibiting reactive oxygen species. A considerable number of natural extracts proved to possess neuroprotective activity [10, 11]. There is evidence that seizures lead to neuronal loss and brain damage. Moreover, death of cell in discrete brain regions is considered as one of the characterizing anatomical features of epilepsy [12]. Brain regions that are more vulnerable to seizure disorders include the amygdala and the hippocampus in addition to the neighboring piriform and entorhinal cortex [13, 14]. The role of oxidative stress and free radicals in seizure disorders has been documented extensively [15, 16]. Pentylenetetrazole (PTZ) is chemical toxin reported to interact with GABA-A receptor complex in the adult brain [17]. It can reach the brain through successful transportation via blood-brain barrier [18]. Furthermore, animal data suppose that even a single seizure can have damaging effect and may lead to fragmentation of DNA; a feature characterizing apoptosis.* Phragmanthera austroarabica *(Loranthaceae) is a semiparasitic plant collected from Saudi Arabia. To date, there is no information about the effect of* P. austroarabica* extract on animal models of neurologic disorders and epilepsy. So, this study was performed to examine the possible neuroprotective effect of the extract in PTZ-kindling model in mice. Previous chemical study of the plant led to isolation and chemical identification of twelve compounds. Ten out of the twelve compounds exhibited potent free radical scavenging property when tested against DPPH reagent. These compounds, namely, are chrysophanic acid and its 8-*O*-glucoside, emodin and its 8-*O*-glucoside, methyl gallate, pectolinarigenin, quercetin, catechin and its 4′-*O*-gallate, and dillenetin-3-*O*-glucoside. Additionally, the total plant extract revealed significant antioxidant effect [19]. In the present work, two compounds (gallic acid and *β*-sitosterol-3-*O*-glucoside) were identified. In addition, the extract was standardized where total phenolic contents were assessed by the use of Folin-Ciocalteu reagent.

## 2. Materials and Methods

### 2.1. In Vivo Determination of Anticonvulsant and Neuroprotective Activity

#### 2.1.1. Animals and Housing Conditions

Thirty albino male mice were used in this study with access to water and food. Mice were housed in polyethylene cages under a normal light/dark cycle and allowed to acclimatize for the experiment conditions for one week before starting of drug injection. Seizure scoring was done at 14:00–17:00 h to reduce influence of circadian on seizure susceptibility. All possible efforts were done to reduce animals suffering. Study protocol was approved by the committee of research ethics at Faculty of Pharmacy in Suez Canal University* (License number 20146A7)*.

#### 2.1.2. Pentylenetetrazole-Induced Kindling in Mice

Pentylenetetrazole was obtained from Sigma-Aldrich (MO, USA) and then prepared by dissolving in sterile saline. For induction of kindling, mice were injected intraperitoneally with PTZ (35 mg/kg) trice a week to reach a total number of thirteen injections [20] to produce the model of chemically induced chronic epilepsy. Mice in the vehicle group were treated by injection with saline parallel to PTZ injection.

#### 2.1.3. Study Groups

Thirty mice were divided into five groups in a random way, six mice each as follows:* Group I (control saline group)*: injection by saline was done every second day to reach a total of 22 injections.* Group II (PTZ control group)*: mice were injected with subconvulsive doses of PTZ (30 mg/kg, i.p.) every second day for a total of 22 injections [21].* Groups III–V (PTZ + P. austroarabica groups)*: mice were injected with PTZ as mentioned above and on the same days, they will receive protective doses of the extract (200, 400, or 800 mg/kg, p.o.), respectively, 45 min before each PTZ injection. The total number of protective doses was 22. Importantly, the selected doses of the extract were determined according to those reported in previous studies either for the same plant or for closely related plants belonging to the same family [22–25].

#### 2.1.4. Evaluation of Seizure Activity in PTZ-Kindled Mice

The observation of convulsive behavior was done along 30 min in each mouse after PTZ injection. The severity of the seizures was ranked based on Racine scale [26] which is commonly used for assessment of seizure activity in rodents [27]. Mice were scored as follows: (0): if there is no seizure response, (1): if there exist immobility, closure of the eye, twitching of the ear, and appearance of facial clonus, (2): if there is nodding of head together with highly severe facial clonus, (3): if there is clonus of one forelimb, (3, 5): if there is bilateral forelimb clonus and no rearing, (4): if there is bilateral forelimb clonus accompanied with rearing, (4, 5): if there is no rearing and animals fall on a side, and righting reflex was lost in addition to generalized tonic-clonic seizures, and (5): if rearing occurred and animals fell on back together with generalized tonic-clonic seizures. After the last PTZ injection, an average of thirteen seizure scores recorded throughout the course of the experiment in each experimental group were calculated and compared. In addition, the percent of mice survival in each experimental group was determined.

#### 2.1.5. Dissection of Brain and Processing of the Two Hemispheres

After completing the experiment, the mice were anaesthetized using thiopental sodium (50 mg/kg) and then were sacrificed by cervical dislocation. This was followed by removal of brains and one hemisphere was frozen immediately. The frozen hemisphere was used for isolation of the cortex and hippocampus. Those were used for DNA laddering assay and calculation of oxidative stress parameters. Other hemisphere was fixed for 24 h in 10% paraformaldehyde solution. This was followed by cutting of the brain into sections on a vibratome. Two coronal sections at the same hippocampal level were stained with hematoxylin and eosin (H&E) or Cresyl violet stain and protected with a cover-slip; the stain helps to declare the cytoarchitecture of cortex and hippocampus and to evaluate cell degeneration.

#### 2.1.6. Determination of Oxidative Stress Parameters

The cortex and hippocampus from each brain were isolated while frozen. In order to measure oxidative stress parameters, one part of tissues was homogenized in one ml of phosphate-buffered saline (pH = 7.4) by the use of a Teflon homogenizer (Glass Col homogenizer system, Vernon hills, USA). Centrifugation of the tissue homogenate was performed at 4°C at 3000 ×g for 15 min; this step was followed by collection of the supernatant to be used in the different assays. Homogenates were assayed for malondialdehyde (MDA) and reduced glutathione (GSH) using commercial colorimetric kits obtained from Biodiagnostic Company (Giza, Egypt). Instructions reported by the manufacturer were followed. MDA was measured according to the methods based on its reaction with thiobarbituric acid (TBA) in an acidic medium and temperature equals 95°C for 30 minutes. The yielded product is TBA-reactive. Measurement of absorbance of the reaction product was done at 534 nm as reported previously. Tissue GSH was estimated using the method depending on the reduction of 5,5′-dithiobis(2-nitrobenzoic acid) (DTNB) with reduced GSH to form a yellow colored product. This color is directly proportional to GSH concentration. Absorbance was determined colorimetrically by measurement at 405 nm.

#### 2.1.7. Extraction of DNA and DNA Ladder by Gel Electrophoresis

Assessment of endonuclease-dependent ladder-like DNA fragmentation was done. Gel electrophoresis technique was used. Extraction of genomic DNA from the brain tissues was achieved by the use of Bio Basic EZ-10 spin column genomic DNA kit (Markham, Canada), following the manufacturers protocol. The samples of extracted genomic DNA were subjected to electrophoresis by the use of 0.8% (w/v) agarose gel at 90 V and 110 mA (2 h). After that, samples were stained using ethidium bromide; this was followed by visualization under UV light. The 100 bp DNA ladder kit (Solis Biodyne, Tartu, Estonia) is a molecular weight marker for ready use. Photos for the gel were captured by the aid of gel documentation system and interpreted using Gel Docu advanced ver. 2 software.

#### 2.1.8. Quantification of Surviving Neurons in the Cortex and Hippocampus of Rats

The solution used for staining consisted of Cresyl violet (0.5 g) in distilled water (100 ml). Removal of the matrix was done by washing of microtome sections by tap water, followed by their dipping three times in distilled water. This step was followed by staining at room temperature (for ten minutes) using Cresyl violet stain dye and then drying in air for one hour. The dry sections were dipped shortly in alcohol. Sections were cleared using xylene and protected using Entellan cover-slip. Finally, they were microscopically checked.

Cresyl violet staining was performed for staining of Nissl's substances, which are the clumps of ribosomes associated with membrane of endoplasmic reticulum in the nerve cells [28]. The method depends on detection of Nissl substance in sections of the tissue which are fixed with formalin and embedded in paraffin. Staining is usually used for identification of basic neuronal structure within the brain [29]. The cortex and hippocampus were chosen since they are the most sensitive area to toxic insults and important for maintaining epileptic seizures. Quantification of the surviving cells was performed within the cortex and hippocampus from all animals cut at the same level.

Neuronal morphology was performed in ten regularly spaced sections among the entire surface of cortex and hippocampus. Counting of the neurons was based on identification of a clear and distinct nuclear membrane. The only neurons considered viable are those with visible nuclei and with the entire outline of the cell appearing complete. The mean of viable neurons in the ten regularly spaced sections was calculated for each mouse in each group [30, 31] and calculated as % from the total number of the cells. The numbers of cells in each of the cortical or hippocampal areas are as those counted from photographs captured at 40x magnification [32–34].

Pycnotic cells in different hippocampal area were counted. Identification of the dead cells was done by morphological criteria by the blebbing in plasma membrane and shrunken cytoplasm, surrounded by perineuronal vacuoles, neuron size alteration, and triangular shape of the cells. For microscopic evaluation, ten sections covering the entire surface of the cortex and hippocampus were analyzed. Then, the sum of cell counts from the ten sections was calculated as % from the total number of cells and then averaged for each animal.

#### 2.1.9. Statistical Analyses

The data taken from this study were tabulated and expressed as mean ± standard error. One-way analysis of variance was used for analysis of the results, followed by Bonferroni's post hoc test. The percent of surviving mice was compared applying the Chi square test. The statistical package for social science, version 16 (SPSS Software, SPSS Inc., Chicago, USA) was employed for this purpose. All *P* values were two-tailed and *P* < 0.05 was considered statistically significant.

### 2.2. Phytochemical Study

#### 2.2.1. General Experimental Procedures

Nuclear magnetic resonance (NMR) spectra were obtained in CD_3_OD on Bruker Avance DRX 600 spectrometers at 600 MHz for hydrogen NMR (^1^HNMR) and 150 MHz for carbon NMR (^13^C NMR). For separation by column chromatography, silica gel (Merck, 70–230-mesh ASTM) and Sephadex LH-20 (Pharmacia) were utilized. Further, precoated silica gel 60 F-254 plates (Merck) were used for thin-layer chromatography (TLC). Spots were visualized by exposure to NH_3_ vapour, UV radiation, and *P*-anisaldehyde/sulfuric acid.

#### 2.2.2. Plant Material

Collection of the plant was done in March (2011) from Abha, Khamis Mushait at South Saudi Arabia. Identification of the plant was confirmed by Dr. Nahed Morad (Faculty of Science at King Abdulaziz University). A specimen was deposited at Natural Products Department, Faculty of Pharmacy, King Abdulaziz University, with a code number 2011-Phaa.

#### 2.2.3. Chemical Study

The air dried plant was powdered (0.5 kg) and macerated with methyl alcohol (2 × 1000 mL), concentrated, and fractionated using hexane, chloroform, and ethyl acetate, respectively. Two grams of chloroform extract was fractionated on a silica gel column. Then, the extract was eluted with chloroform : methanol gradient. After that, fractions eluted by methanol : chloroform (3 : 97) were further fractionated on a silica gel column with gradient elution using chloroform-methanol, followed by repeated crystallization from methanol to give 15 mg of a white powder (A). Four grams of the ethyl acetate extract was chromatographed on a column packed with silica gel and then eluted with chloroform : methanol gradient. Fractions eluted by 20% methanol in chloroform were then purified on Sephadex, eluted with methanol to give 65 mg of creamy powder (B).

#### 2.2.4. Assessment of Total Phenolic Content

Total phenolic contents were determined by spectrophotometry, using gallic acid as a standard (prepared to cover the range of 0–200 mg%). Briefly, 20 mg of the dry extract was dissolved in 15 ml methanol; the volume was completed to 20 ml using methanol. Extract (0.1 mL) was mixed with distilled water (2.8 ml), 2% sodium carbonate solution (2 ml), and Folin-Ciocalteu's reagent (0.1 ml). The mixture was left for 30 minutes. Absorbance was measured at 750 nm against distilled water blank [35].

## 3. Results

### 3.1. Effect of* P. austroarabica* Extract on Seizure Activity in Pentylenetetrazole-Kindled Mice

In the current study, repetitive injection of PTZ resulted in kindling in mice. Mice showed progressively increased seizure scores and were compared at the final score. The final seizure score exhibited by mice in the PTZ control group was greater than that exhibited by the saline group (4.33 ± 0.31 versus 0 ± 0, *P* < 0.05, Figure 2). Treatment with diazepam significantly reduced the final seizure score compared to the PTZ control group. Treatment with* P. austroarabica* extract (200 mg/kg) did not significantly ameliorate the final seizure score in comparison to PTZ control group. However, the middle and high doses of* P. austroarabica* extract (400 or 800 mg/kg) significantly reduced the final seizure score compared to PTZ group (2.6 ± 0.22 or 2 ± 0.29 versus 4.2 ± 0.34, resp., *P* < 0.05, Figure 2). Importantly, the score recorded in mice treated with* P. austroarabica* extract (800 mg/kg) was not significantly different from that recorded in mice treated with diazepam.

### 3.2. Effect of* P. austroarabica* Extract on Cortical Malondialdehyde and Reduced Glutathione and Integrity of Cortical DNA Pentylenetetrazole-Kindled Mice

The results of the current study indicated that PTZ control group showed lower cortical GSH and greater MDA level compared to saline group. Treatment with* P. austroarabica* extract (200 or 400 mg/kg) did improve cortical GSH level; however, the higher dose of the extract (800 mg/kg) enhanced cortical GSH level compared to PTZ control group (*P* < 0.05, Figure 3(a)). Regarding cortical MDA level, treatment with* P. austroarabica* extract (400 or 800 mg/kg) reduced the detected level in comparison to the PTZ control group (Figure 3(b)). Furthermore, gel electrophoresis for DNA samples from the study groups demonstrated high degree of fragmentation in PTZ control group. Meanwhile, mice treated with* P. austroarabica* extract (200 mg/kg) did not improve the quality of the DNA ladder (Figure 4).

### 3.3. Effect of* P. austroarabica* Extract on Survival of Cortical Neurons in Pentylenetetrazole-Kindled Mice

Figures 5(a)–5(e) are photographs for sections from the cortex of different experimental groups stained with Cresyl violet stain.  Figure 5(f) indicates that PTZ control rats showed lower percent of surviving neurons compared to saline group. Treatment with the highest dose of* P. austroarabica* extract (800 mg/kg) increased the percent of surviving neurons compared to PTZ control group (Figure 5(f)).

### 3.4. Effect of* P. austroarabica* Extract on Survival of Hippocampal Neurons in Pentylenetetrazole-Kindled Mice

Figures 6(a)–6(e) demonstrate photographs for the hippocampus of different study groups showing different degrees of staining with Cresyl violet stain. In Figure 6(f), the mean percent of surviving neurons showed that PTZ control group exhibited lower percent of surviving neurons in comparison to the saline group. Furthermore, administration of* P. austroarabica* extract (800 mg/kg) enhanced the percent of surviving neurons in comparison to PTZ control group (Figure 6(f)).

### 3.5. Effect of* P. austroarabica* Extract on Pyknosis in Cortical Neurons in Pentylenetetrazole-Kindled Mice

Figures 7(a)–7(e) show some photographs for cortical sections stained with H&E from the different experimental groups. Pyknosis appears in the form of shrunken neurons with dark cytoplasm surrounded by vacuoles in most of cases.  Figure 7(f) represents the mean of percent of pyknotic neurons in each group and highlights that PTZ-kindled mice show greater percent of pyknotic neurons in comparison to the percent registered in saline group. Treatment with the high dose of* P. austroarabica* extract (800 mg/kg) resulted in a reduction in the percent of pyknotic cortical neurons compared to PTZ group, but lower doses did not result in a similar effect.

### 3.6. Effect of* P. austroarabica* Extract on Pyknosis in Hippocampal Neurons in Pentylenetetrazole-Kindled Mice

Figures 8(a)–8(e) demonstrate photographs for the hippocampal neurons in the study groups. The PTZ-kindled group showed greater percent of pyknotic neurons in comparison to the saline group. Treatment with the moderate or high doses of* P. austroarabica* extract (400 or 800 mg/kg) reduced the percent of pyknotic neurons compared to PTZ-kindled mice (Figure 8(f)).

### 3.7. NMR Data of the Isolated Compounds

*Compound A (β-Sitosterol-3-O-glucoside)*. ^13^C-NMR (150 MHz, CDCl_3_ + CD_3_OD): 37.8 (C-1), 29.7 (C-2), 78.1 (C-3), 39.9 (C-4), 139.8 (C-5), 121.7 (C-6), 32.2 (C-7), 31.7 (C-8), 49.7 (C-9), 37.3 (C-10), 21.6 (C-11), 38.2 (C-12), 50.8 (C-13), 56.2 (C-14), 26.1 (C-15), 25.3 (C-16), 56.1 (C-17), 12.2 (C-18), 19.3 (C-19), 36.1 (C- 20), 19.4 (C-21), 33.9 (C-22), 26.2 (C-23), 46.1 (C-24), 30.4 (C-25), 21.1 (C-26), 21.2 (C-27), 23.2 (C-28), 12.1 (C-29), 102.3 (C-1′), 74.1 (C-2′), 76.3 (C-3′), 70.9 (C-4′), 77.7 (C-5′), 61.9 (C-6′).

*Compound B (Gallic Acid)*. Creamy powder, ^1^H NMR (600 MHz, CD_3_OD): *δ*_H_ 7.02 (2H, s, H-2,6); ^13^C NMR (150 MHz, CD_3_OD): *δ*_C_ 110.1 (C-2,6), 120.0 (C-1), 139.1 (C-4), 145.6 (C-3,5), 168.6 (C=O).

### 3.8. Total Phenolic Contents

Gallic acid was used as a standard for determination of total phenolic content. The total phenolic content in the plant extract was determined and expressed as mg gallic acid equivalents per 1 g of the dry powdered extract. The method was repeated three times and the relative standard deviation was determined where the total phenolic contents were assessed as 379.92 ± 1.32 mg gallic acid equivalents/g dry plant extract. The linearity range was determined as 4–80 mg/ml. The regression equation was as follows: *y* = 0.0083*x* + 0.071 (*y* = the absorbance, *x* = the concentration); correlation coefficient equals 0.9987.

## 4. Discussion

**Figure 1:**
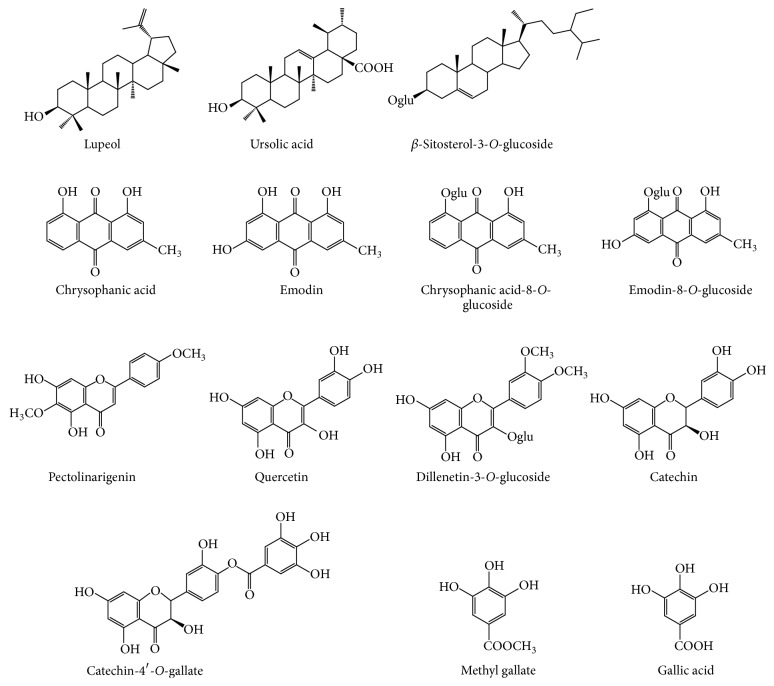
Structure of the compounds of* Phragmanthera austroarabica*.

**Figure 2:**
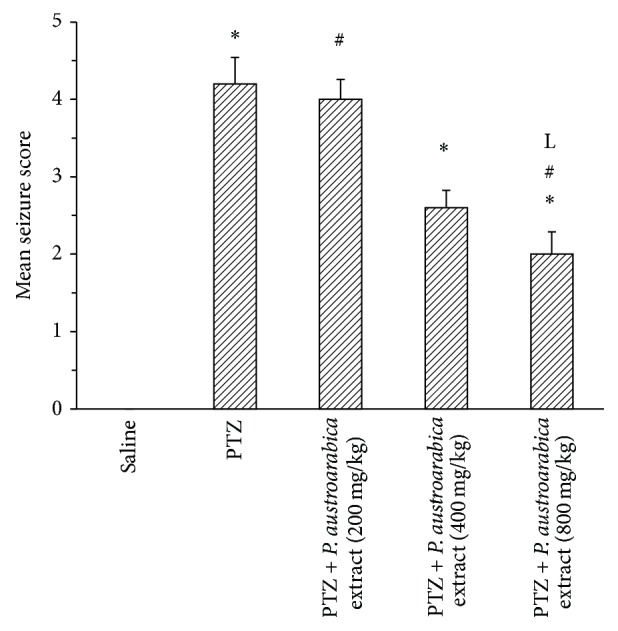
Mean seizure score in the experimental groups. Mice were injected with PTZ (35 mg/kg) trice a week for a total of 13 injections. Seizures were scored (0) if there is no seizure response, (1) if immobility, eye closure, ear twitching, or facial clonus appears, (2) if head nodding associated with more severe facial clonus, (3) if there is clonus of one forelimb, (3, 5) if there is bilateral forelimb clonus without rearing, (4) if there is bilateral forelimb clonus with rearing, (4, 5) if animals fall on a side (without rearing), loss of righting reflex with generalized tonic-clonic seizures, and (5) if rearing and falling on back occur accompanied by generalized tonic-clonic seizures. Results are mean ± SEM and analyzed using one-way ANOVA followed by Bonferroni's post hoc test at *P* < 0.05. ^*∗*^Significant difference from saline group. ^#^Significant difference from PTZ group. ^L^Significant difference from PTZ +* P. austroarabica* extract (200 mg/kg) group, *n* = 4–6.

**Figure 3:**
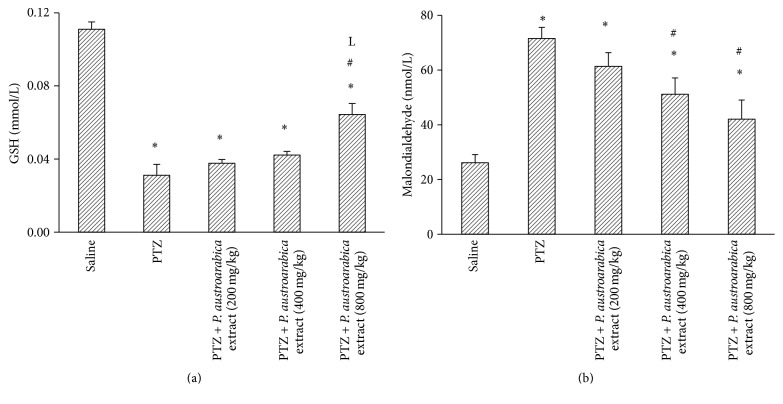
Cortical level of reduced glutathione and malondialdehyde in the experimental groups. Mice were injected with PTZ (35 mg/kg) trice a week for a total of 13 injections. Cortices from frozen brains were homogenized and assayed for reduced glutathione (GSH) and malondialdehyde. Results are mean ± SEM and analyzed using one-way ANOVA followed by Bonferroni's post hoc test at *P* < 0.05. ^*∗*^Significant difference from saline group. ^#^Significant difference from PTZ group. ^L^Significant difference from PTZ +* P. austroarabica* extract (200 mg/kg) group, *n* = 4–6.

**Figure 4:**
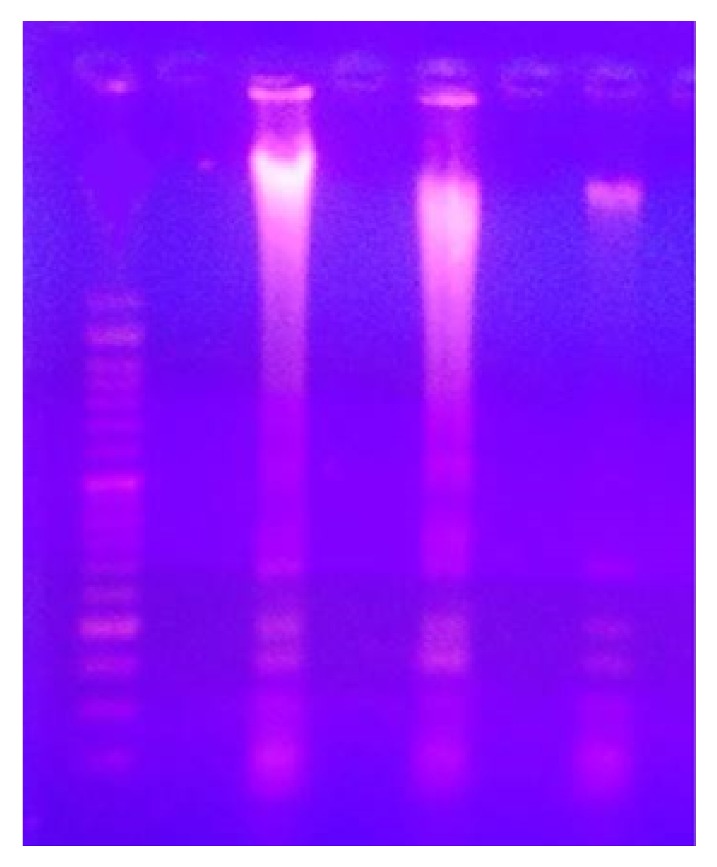
Agarose gel electrophoresis for DNA samples from the experimental groups. DNA was extracted from the cortical homogenate from one frozen brain hemisphere and loaded 100 bp ladder. The first lane is showing smeared DNA from one mouse in PTZ control group. The second lane shows DNA from PTZ +* P. austroarabica* extract (200 mg/kg) group showing internucleosomal DNA fragmentation with mixed smearing. The third lane shows DNA sample from one mouse in PTZ +* P. austroarabica* extract (800 mg/kg) with no laddering. DNA samples were run over agarose gel.

**Figure 5:**
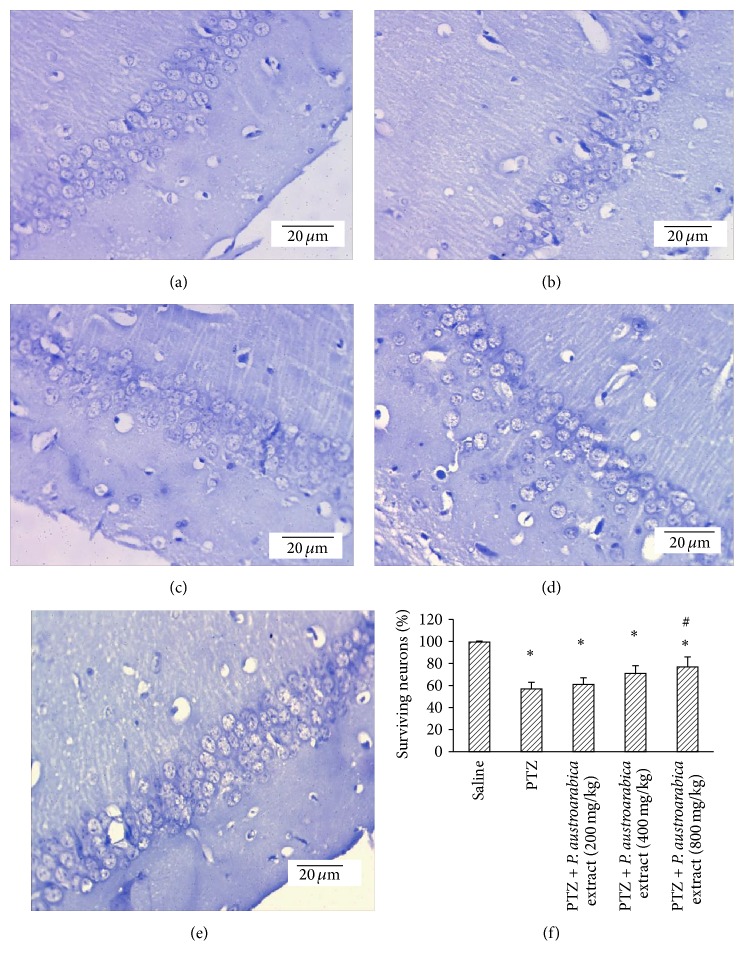
Surviving neurons in cortex of the experimental groups. ((a)–(e)) Photographs for cortical sections from the experimental groups stained with Cresyl violet stain. (f) Mean percent of surviving neurons in the study groups. Mice were injected with PTZ (35 mg/kg) trice a week for a total of 13 injections. Cortices from formalin-fixed brains were cut and stained. Results are mean ± SEM and analyzed using one-way ANOVA followed by Bonferroni's post hoc test at *P* < 0.05. ^*∗*^Significant difference from saline group. ^#^Significant difference from PTZ group.

**Figure 6:**
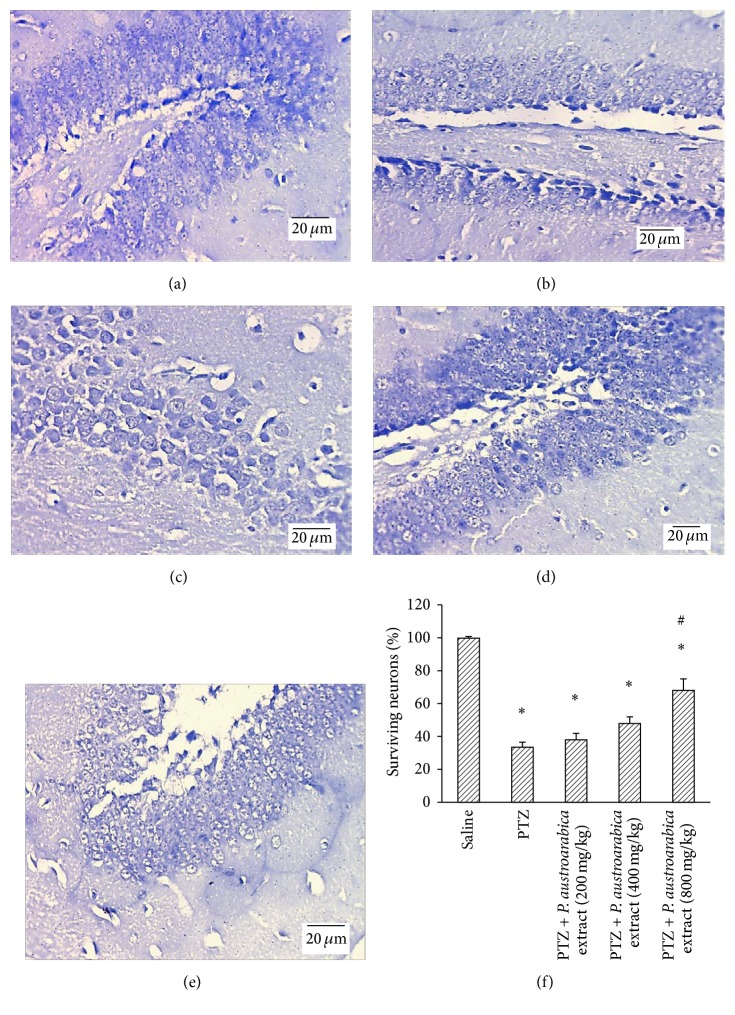
Surviving neurons in hippocampus of the experimental groups. ((a)–(e)) Photographs for hippocampal sections from the experimental groups stained with Cresyl violet stain. (f) Mean percent of surviving neurons in the study groups. Results are mean ± SEM and analyzed using one-way ANOVA followed by Bonferroni's post hoc test at *P* < 0.05. ^*∗*^Significant difference from saline group. ^#^Significant difference from PTZ group.

**Figure 7:**
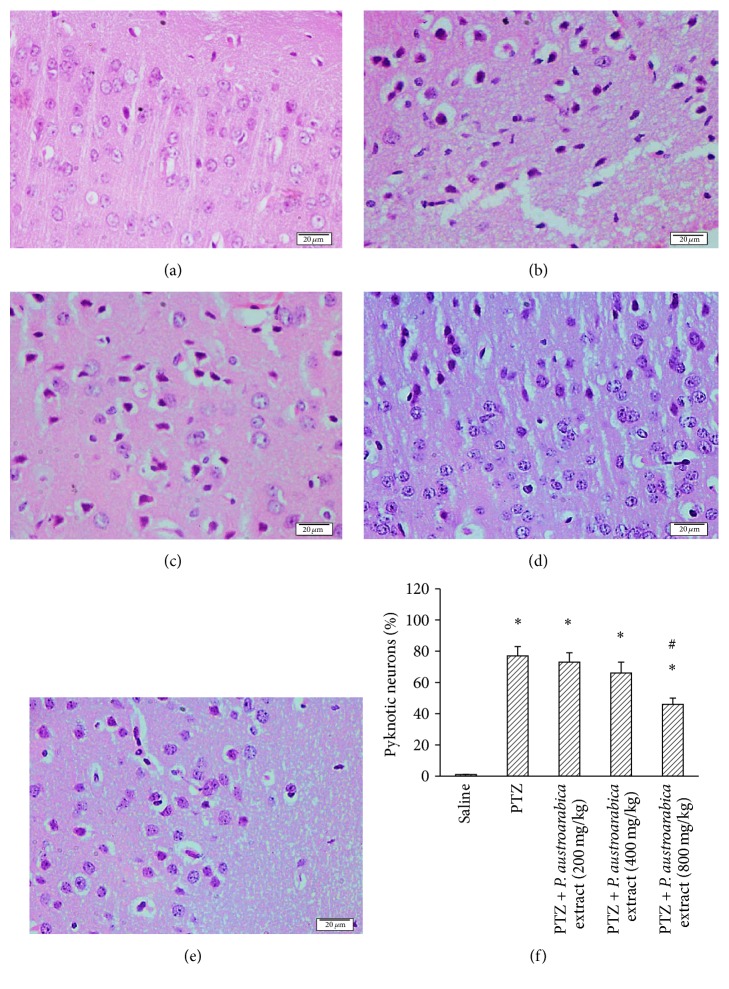
Pyknotic neurons in cortex of the experimental groups. ((a)–(e)) Photographs for cortical sections from the experimental groups stained with hematoxylin and eosin stain. (f) Mean percent of pyknotic neurons in the study groups. Mice were injected with PTZ (35 mg/kg) trice a week for a total of 13 injections. Cortices from formalin-fixed brains were cut and stained. Results are mean ± SEM and analyzed using one-way ANOVA followed by Bonferroni's post hoc test at *P* < 0.05. ^*∗*^Significant difference from saline group. ^#^Significant difference from PTZ group.

**Figure 8:**
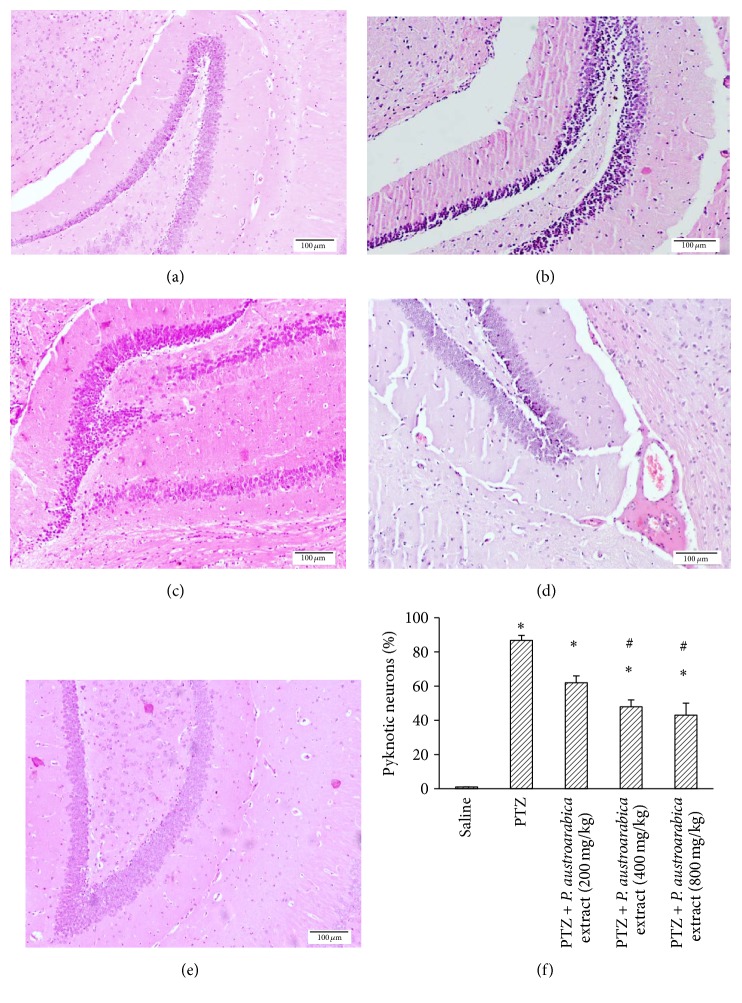
Pyknotic neurons in hippocampus of the experimental groups. ((a)–(e)) Photographs for hippocampal sections from the experimental groups stained with hematoxylin and eosin stain. (f) Mean percent of pyknotic neurons in the study groups. Results are mean ± SEM and analyzed using one-way ANOVA followed by Bonferroni's post hoc test at *P* < 0.05. ^*∗*^Significant difference from saline group. ^#^Significant difference from PTZ group.

The total alcoholic extract of the plant was subjected to different chromatographic techniques as described. Fourteen compounds were detected in this plant, of which twelve were previously identified [19]. Additionally, two compounds were isolated and chemically identified as *β*-sitosterol-3-*O*-glucoside and gallic acid. The two compounds are known and previously reported from different plants. Structure elucidation of the compounds was based on different NMR data in addition to comparison with the data previously reported in literature [36, 37]. Other compounds were identified by direct comparison using TLC with those previously isolated by the author from the same plant. The chemical constituents of* Phragmanthera austroarabica* are illustrated in Figure 1.

The total phenolic content was found to be 379.92 ± 1.32 mg gallic acid equivalents/g dry plant extract. The validity of the method was proved by the small value of the correlation coefficient (0.9987).

In the current study, repeated injection of PTZ produced a gradual increase in seizure activity during the course of the experiment. This activity was accompanied by oxidative stress, as indicated by high cortical malondialdehyde content and lower percent of surviving neurons in the cortex and hippocampus. The current results declared that the hippocampus was greatly affected by PTZ-kindling compared to the cortex. Many studies reported that repeated injection of subconvulsive doses of PTZ increases the susceptibility of the animals, resulting in a fully kindled state. In this case, brief seizures last for few minutes resembling human epilepsy along with histopathological abnormalities in some brain regions [20]. It is well known that seizures result in and enhance brain damage [14, 32]. For this reason, the kindling model offers a good tool for studying the pathophysiological mechanisms and cumulative changes during epileptogenesis [38]. Biological testing highlighted that the medium and high doses of* P. austroarabica* extract ameliorated seizure activity as indicated by low seizure scores in PTZ-kindled mice. This is the first report to demonstrate the central activity of this extract. However, amelioration of the seizure activity does not necessarily imply a direct anticonvulsant activity for the extract, but this ameliorative effect may arise from other biological activities such as suppression of oxidative burden. Further, some antioxidant properties were outlined by increasing cortical GSH and reducing MDA level upon treatment with the high dose of the extract. The antioxidant activity may contribute to the observed effect of the extract as similar properties were documented previously in animal models of diabetes [23]. Extensive studies for experimental models reported that continuous seizures or even repetitive brief seizures in kindling provoke pathological neurodegeneration in the brain [39, 40]. At the cellular level, the high dose of the plant extract provided beneficial neuroprotective effect according to the increase of the percent of surviving neurons in the cortex and hippocampus. It is also important to note that the high dose is mostly the dose that produced biological activity. This makes it necessary to determine the safety margin of this extract.

Actually, only two articles were detected in the literature concerning the biological effect of this extract. These reported studies described the beneficial effects of* P. austroarabica *extract in treating experimental diabetes [23]. It is worth mentioning that previous studies were conducted on pure compounds that are present as major constituents in this plant. These studies exhibited their beneficial effect as neuroprotective agents. Among these compounds are quercetin [41], gallic acid [42, 43], catechin [44], and emodin [45]. Additionally, many phenolic compounds were previously reported as neuroprotective [46], whereas* P. austroarabica *extract is considered to accumulate a considerable amount of phenolic constituents. These facts can additionally justify the efficacy of* P. austroarabica *extract as neuroprotective in the studied models in our study.

## 5. Conclusion

In conclusion, the current study represents a starting point for more research about the neuroprotective effect of* P. austroarabica *extract in various models of epilepsy, neurotoxicity, and neurodegenerative diseases and further elucidates the detailed mechanism of action.
